# The Impact of Long-Term Care Insurance Payment Modes on Healthcare Utilization and Expenditures Among Middle-Aged and Older Adults in China

**DOI:** 10.3390/healthcare14091157

**Published:** 2026-04-25

**Authors:** Xinfang Li, Mingqiang Li, Zhihui Li

**Affiliations:** 1 Dong Fureng Institute of Economic and Social Development, Wuhan University, Wuhan 430072, China; 2 Taikang Era of Longevity Institute, Taikang Insurance Group Co., Ltd., Beijing 100026, China; 3 Vanke School of Public Health, Tsinghua University, Beijing 100084, China; zhihuili@tsinghua.edu.cn

**Keywords:** long-term care insurance, benefit payment modes, healthcare utilization, medical expenditures, DID (difference-in-differences) model

## Abstract

**Objectives**: This study examines how different benefit payment modes under China’s long-term care insurance (LTCI) program influence healthcare utilization and medical expenditures among middle-aged and older adults. Specifically, it compares the effects of in-kind benefits and mixed benefits on healthcare service use and financial burden. **Methods**: This study uses data from the China Health and Retirement Longitudinal Study (CHARLS) from 2011 to 2018, focusing on middle-aged and older adults with functional limitations. Exploiting the staggered implementation of LTCI pilot programs across 14 cities, a difference-in-differences (DID) approach is employed to estimate the causal effects of different benefit payment modes on healthcare utilization and expenditures. Heterogeneity analyses are conducted to explore differences between rural and urban populations. **Results**: The results indicate that the in-kind benefit mode significantly reduces inpatient visits, total medical costs, and out-of-pocket expenditures. By contrast, the mixed benefit mode shows only a modest reduction observed mainly in outpatient visits. Heterogeneity analysis further reveals that in-kind benefits are particularly effective in reducing healthcare utilization and medical expenditures among rural residents, while urban residents experience higher reductions in out-of-pocket spending. **Conclusions**: These findings highlight the importance of benefit design in shaping the effectiveness of LTCI policies. Prioritizing service-based benefits may improve healthcare system efficiency and reduce financial burdens among older adults. The results provide policy-relevant insights for optimizing LTCI benefit design in China and other aging societies.

## 1. Introduction

Population aging is accelerating worldwide. Global data indicate that the share of people aged 65 and over will nearly triple between 2000 and 2050. Asia is projected to see one of the fastest-growing older populations globally, with China experiencing especially rapid population aging [1,2,3]. According to the Seventh National Population Census, by the end of 2020, China had 260 million people aged 60 and over (18.7% of the total population), including about 190 million aged 65 and over—more than one-fifth of the world’s population in this age group. Meanwhile, the number of older adults living with disabilities has risen sharply; data from the China National Committee on Aging show that over 42 million people aged 60+ were living with disabilities in 2020, accounting for approximately 16.6% of the older population [4]. The large size of this vulnerable population places additional pressure on healthcare services and financial systems, as older adults, particularly those with disabilities, tend to have higher healthcare utilization and face a larger burden of out-of-pocket medical expenditures [5,6].

Against the backdrop of a declining capacity for informal family care, driven by shrinking household sizes, lower rates of intergenerational co-residence, increased female labor force participation, and rapid urbanization, the establishment of an institutionalized long-term care insurance (LTCI) system has emerged as a key policy response to population aging in China [7,8]. As an integral component of the social security system, LTCI aims to mitigate the financial risks associated with long-term care needs and to improve access to essential healthcare services among older adults [9,10]. Internationally, countries such as Germany, Japan, and South Korea have implemented LTCI systems for many years, adopting diverse benefit payment models, including in-kind benefits, cash benefits, and mixed benefit arrangements, in accordance with their demographic and socioeconomic contexts [11,12,13,14,15,16]. Evidence from these countries indicates that benefit payment design is a key determinant of LTCI effectiveness in improving healthcare access and reducing financial burdens.

Despite the rapid development of LTCI in China, the empirical evidence on the comparative effectiveness of different benefit payment modes remains limited. Existing studies have mainly focused on the overall impact of LTCI on healthcare utilization or expenditures [17,18,19,20,21,22,23], yet few have systematically examined the heterogeneous effects of in-kind versus mixed benefit models, particularly among China’s aging population, which has unique healthcare needs. In-kind benefit mode provides only care services to beneficiaries, whereas the mixed mode offers beneficiaries a choice between services and cash subsidies. This research gap is critical because the choice of benefit payment mode directly affects the targeting efficiency of LTCI: in-kind benefits can ensure that resources are allocated to essential healthcare services, whereas mixed benefits, while offering greater flexibility, carry the risk of cash subsidies being diverted to non-medical expenditures [24,25]. For China’s elderly population, many of whom face financial constraints, limited health literacy, or inadequate access to primary healthcare, identifying which benefit mode is more effective in improving healthcare utilization and reducing out-of-pocket burdens is of strong significance for optimizing LTCI policy design.

Against this backdrop, we investigate how different LTCI benefit payment modes affect healthcare utilization and medical expenditures among middle-aged and older adults in China. Rather than examining the overall impact of LTCI participation, this study focuses on the heterogeneous effects of in-kind and mixed benefit models. Using a difference-in-differences (DID) approach, this paper examines the impact of different benefit payment modes, including in-kind versus mixed, on healthcare utilization and financial burden in China. Our analysis considers two key outcome dimensions: healthcare utilization, measured by inpatient and outpatient visit frequencies, and healthcare expenditures, including total medical costs and out-of-pocket expenses. This study tests three specific hypotheses: (1) In-kind LTCI benefits reduce inpatient utilization and out-of-pocket expenditures more effectively than mixed benefits. (2) The overall impact of LTCI on medical spending is driven primarily by the in-kind model. (3) The effects of benefit payment modes differ by residence, with in-kind benefits yielding larger reductions in healthcare use among rural residents and higher out-of-pocket savings for urban residents. Our findings show that in-kind benefits consistently reduce inpatient visits, total medical expenditures, and out-of-pocket costs, whereas mixed benefits yield only modest and limited effects—primarily on outpatient use. Moreover, these impacts vary by residence: in-kind benefits are especially effective at curbing healthcare use among rural residents, while urban beneficiaries experience larger reductions in out-of-pocket spending.

While a growing body of evidence documents the aggregate effects of China’s LTCI pilots on healthcare utilization and expenditure, existing studies largely treat the program as a uniform intervention. This aggregation obscures a fundamental dimension of policy heterogeneity: benefit payment mode. In-kind benefits directly channel resources into formal care services, creating a substitution pathway between long-term care and medical utilization. Mixed benefits, by contrast, partially transfer cash to beneficiaries or informal caregivers, introducing behavioral flexibility that may dilute the program’s impact on formal healthcare use. These two modes operate through distinct causal mechanisms and are therefore likely to produce divergent effects on healthcare utilization and expenditure. By providing empirical evidence on how different benefit payment modes influence healthcare utilization and financial burden, the study makes three contributions. First, it provides a systematic causal comparison of in-kind versus mixed benefit designs in LTCI, shifting the focus from whether LTCI works to which design works better. Second, it shows that in-kind benefits consistently reduce inpatient visits, total healthcare costs, and out-of-pocket spending, while mixed benefits have only limited effects. We offer direct evidence on which benefit structure is more effective at achieving the primary fiscal and utilization goals of LTCI. Third, it reveals urban–rural differences: in-kind benefits are especially effective for rural residents, whereas urban residents see larger out-of-pocket savings, highlighting that the optimal benefit design depends on the local context, a nuance missed by aggregate program evaluations and highly relevant for China’s ongoing national LTCI expansion. The findings also offer policy-relevant insights for optimizing LTCI benefit design, improving access to healthcare services, and enhancing the well-being of aging populations in China and other countries facing similar demographic challenges.

## 2. Institutional Background

Since the mid-20th century, several countries have established LTCI systems in response to population aging and its associated health and social care challenges [26,27,28,29]; inspired by these international experiences, China launched its LTCI pilot program in 2016 through the Ministry of Human Resources and Social Security’s “Guiding Opinions on the Pilot Implementation of Long-Term Care Insurance” to address the urgent care needs of disabled older adults, with benefits in pilot regions delivered via three arrangements—institutional care (full-time residence in designated facilities with direct provider reimbursement), home-based care (either professional home visits reimbursed by LTCI or cash subsidies to family caregivers for informal care), and community-based care (provided through outreach or itinerant services, often combined with home visits)—and pilot cities broadly classified into two types: those offering only in-kind benefits and those offering mixed benefits allowing beneficiaries to choose between in-kind services and cash payments. We examined the 14 pilot cities that rolled out LTCI between 2016 and 2017, and the characteristics of their benefit payment modes are summarized in Table 1. According to a report published by the Chinese government on 20 November 2024, approximately 180 million people have been covered by LTCI, with over 2.6 million disabled insured individuals receiving benefits and total fund expenditures exceeding 80 billion RMB. A notable feature of China’s LTCI pilots is the diversity of benefit payment modes, with some cities adopting in-kind benefits and others implementing mixed benefit modes that combine in-kind services with cash subsidies for healthcare expenses. This diversity in benefit payment modes provides a unique quasi-experimental setting to examine how in-kind and mixed benefit designs influence healthcare utilization and out-of-pocket expenditures.

## 3. Materials and Methods

### 3.1. Data

This study uses data from the China Health and Retirement Longitudinal Study (CHARLS), administered by the National School of Development at Peking University. The 2011 baseline survey covers approximately 10,000 households across 150 counties/districts and 450 villages/communities in China, including 17,706 respondents. The research team conducted the baseline survey in 2011 and followed up with subsequent surveys in 2013, 2015, and 2018, ensuring a high-quality and representative sample. CHARLS has been approved by the Biomedical Ethics Review Committee of Peking University (IRB00001052-11015), and all participants provided written informed consent. CHARLS is selected for three main reasons. First, LTCI primarily targets older adults at risk of functional disability, and CHARLS provides a nationally representative sample of individuals aged 45 and above, closely aligning with the policy’s target population. Second, the survey contains detailed information on healthcare utilization and expenditures, allowing a comprehensive assessment of the effects of different LTCI benefit designs on medical service use and out-of-pocket spending. Third, the survey waves, particularly those in 2015 and 2018, coincide closely with the 2016 rollout of LTCI pilot programs, facilitating DID analyses. Furthermore, CHARLS’s broad geographic coverage encompasses most LTCI pilot cities, enhancing the representativeness and validity of the sample. This study uses data from the 2011, 2013, 2015, and 2018 waves. The pre-policy period is defined using combined data from 2011, 2013, and 2015, while the post-policy period is based on the 2018 survey.

The treatment group comprises older adults with mild, moderate, or severe functional disabilities, defined according to three criteria derived from six basic activities of daily living (ADLs): (1) requiring assistance with at least one ADL; (2) reporting difficulty in at least three ADLs; or (3) being unable to complete all six ADLs. These ADL items align with the Barthel Index standard employed by most LTCI pilot cities. Due to differences in response formats in CHARLS (Likert-scale items), the study population comprises individuals with functional limitations, operationalized using the Activities of Daily Living (ADL) measure from CHARLS, which is broader than the formal eligibility criteria used in actual LTCI pilot programs. As a result, our estimates should be interpreted as an “intention-to-treat among potentially eligible” effect. Robustness checks using stricter definitions confirm the stability of our findings [30]. To minimize selection bias, cities that implemented LTCI before 2016 or after 2018 are excluded. The final analytical sample includes 14 pilot cities. We acknowledge that, while the CHARLS data provide multiple pre-policy waves (2011, 2013, 2015), most pilot cities implemented LTCI after the last survey wave (2018), resulting in only one post-policy observation. This limits our ability to examine dynamic treatment effects or assess the evolution of impacts over time.

### 3.2. Variables

Dependent variables. This study examines outcomes across two main dimensions: healthcare utilization and healthcare expenditures. Healthcare utilization includes outpatient visits and inpatient admissions, capturing the intensity of medical service use across different care settings. Outpatient visits are measured as the monthly number of outpatient consultations, while inpatient admissions are measured as the annual number of hospitalizations. Healthcare expenditures are measured using total medical costs and out-of-pocket medical costs. To further distinguish financial burdens across care settings, we also examine outpatient out-of-pocket expenditures and inpatient out-of-pocket expenditures. These outcome variables allow for a comprehensive evaluation of how different LTCI benefit payment modes affect both the intensity of healthcare use and the associated financial burden among middle-aged and older adults. All medical cost outcomes are measured in RMB per year, based on self-reported expenses annually preceding each CHARLS interview wave.

Independent variables. The central explanatory variable in this study is exposure to the LTCI pilot policy, which is identified using a DID framework. Cities in which the LTCI pilot policy is implemented are defined as the treatment group, while cities without LTCI implementation during the study period serve as the control group. Specifically, the treatment indicator (Treat_i_) equals 1 if an individual resides in an LTCI pilot city and meets the eligibility criteria for the program based on health insurance coverage and functional disability status, as measured by activities of daily living (ADL), and 0 otherwise. The post-policy indicator (Post_t_) equals 1 for observations after the implementation of the LTCI pilot policy and 0 for all pre-policy periods.

Control variables. Following the existing literature on healthcare utilization and expenditures among older adults, this study includes a comprehensive set of individual-level control variables to account for demographic characteristics, socioeconomic status, health insurance coverage, and health conditions. Specifically, the control variables comprise age, educational attainment, marital status, household registration type (hukou), enrollment in basic medical insurance, participation in pension insurance, number of children, household consumption per capita, presence of chronic diseases, and recent inpatient experience. Following prior research, we use consumption rather than income as the measure of economic resources, as it is more stable and reliable in the context of developing countries [31]. These covariates help control for observable factors that may simultaneously influence healthcare utilization, medical expenditures, and participation in the LTCI program.

Descriptive statistics. This study uses four waves of CHARLS data (2011, 2013, 2015, and 2018), including individuals from both LTCI pilot and non-pilot cities. As shown in Table 2, this sample size is the sum of observations across the three groups (control, in-kind treatment, and mixed treatment). Descriptive statistics for the main variables are presented in Table 2. Within pilot cities, the mixed benefit group receives a combination of in-kind services and cash benefits, while the in-kind group receives only services. The control group consists of individuals from non-pilot cities who are not covered by LTCI. As shown in Table 2, healthcare utilization and medical expenditures differ across LTCI benefit payment modes. Compared with the in-kind group, beneficiaries in the mixed benefit group report higher outpatient and inpatient utilization and higher total and out-of-pocket medical costs, particularly for inpatient care. Differences in demographic and socioeconomic characteristics across groups are relatively small, suggesting reasonable comparability between treatment and control groups, which provides a foundation for subsequent DID analyses.

### 3.3. Empirical Strategy

This study employs a DID design to estimate the causal effects of different LTCI benefit modalities on healthcare utilization and expenditures among China’s middle-aged and older adults. The DID approach, a standard method for policy evaluation, relies on the parallel trends assumption [32]. This assumption posits that, absent the LTCI intervention, the outcomes for the treatment and control groups would have followed similar trajectories over time. The pre-to-post-policy change in the control group thus provides the counterfactual trend for the treatment group. The causal effect of the policy is then identified by the difference between the actual change in the treatment group (D1) and this counterfactual change (D2), yielding the DID estimator (D1–D2). This method effectively nets out time-invariant confounders and common temporal shocks.

Existing studies have applied the DID approach to examine the effects of LTCI on healthcare utilization and medical expenditures among older adults [33,34]. However, most of this literature has focused on the overall impact of LTCI participation, with limited attention paid to the heterogeneous effects arising from different benefit payment modes. Building on this strand of research, the present study also employs a DID framework to construct a regression model, aiming to identify and compare the effects of alternative LTCI benefit payment modes, namely in-kind benefits and mixed benefits, on healthcare utilization and healthcare expenditures among middle-aged and older adults in China. The DID model is specified as follows:

$$Y_{it}=\beta_{0}+\beta_{1}{Treat}_{i}+\beta_{2}{Post}_{t}+\beta_{3}({Treat}_{i}\times {Post}_{t})+{X^{\prime }}_{it}\gamma +\epsilon_{it}$$

where $Y_{it}$ represents the outcome variable for individual i in year t, including healthcare utilization (outpatient visits, inpatient visits) and healthcare expenditures (total medical costs and out-of-pocket costs). ${\mathrm{Treat}}_{i}$ is a binary indicator equal to 1 if an individual resides in an LTCI pilot city and meets the eligibility criteria for LTCI and 0 otherwise. ${\mathrm{Post}}_{t}$ equals 1 for observations after the implementation of the LTCI pilot (2018) and 0 for the pre-policy period (2011, 2013, and 2015). The treatment indicator is constructed as the interaction between residence in a pilot city and having ADL limitations, reflecting exposure to the policy among those most likely to benefit. The coefficient of interest, $\beta_{3}$, captures the average treatment effect of LTCI participation for the relevant benefit payment mode. $X_{it}$ is a vector of individual-level control variables, including age, gender, education, marital status, hukou status, health insurance, pension participation, number of children, household consumption per capita, presence of chronic diseases, and recent inpatient experience. $\epsilon_{it}$ is the error term. All regression models include individual fixed effects and year fixed effects to control for time-invariant unobserved heterogeneity and common time trends, respectively. Standard errors are clustered at the city level to account for within-city correlation over time.

In summary, this study first presents descriptive statistics to examine whether systematic differences exist in healthcare utilization and healthcare expenditures across individuals covered by different LTCI benefit payment modes and those in non-pilot cities. We then employ a DID framework to identify the causal effects of alternative LTCI benefit payment modes on healthcare utilization and medical expenditures among middle-aged and older adults in China. To ensure the robustness of the empirical results, a series of robustness checks are conducted, including tests of the parallel trends assumption and placebo tests. Finally, this study further conducts heterogeneity analyses to examine whether different LTCI benefit payment modes exhibit systematic differences across population groups.

## 4. Results

### Baseline Regression Results

We employed a DID framework to estimate the differential impacts of the mixed-benefit and in-kind benefit modes of LTCI on healthcare utilization and expenditures among middle-aged and older adults in China. The baseline regression results are presented below, organized by outcome category.

Effects on healthcare utilization. The impacts on outpatient and inpatient service utilization are presented together in Table 3. For outpatient visits, both LTCI benefit modes were associated with a reduction. The mixed-benefit mode showed a coefficient of −0.226 (*p* < 0.1), while the in-kind benefit mode yielded a coefficient of −0.0714 (*p* < 0.1). For inpatient visits, the effects diverged between the two modes. The mixed-benefit mode had a small statistically non-significant positive effect (coefficient 0.0186). In contrast, the in-kind benefit mode was associated with a significant reduction in inpatient visits, with a coefficient of −0.0550 (*p* < 0.05). The pronounced reduction in inpatient visits under the in-kind benefit mode is particularly noteworthy. Inpatient care typically reflects more severe health conditions and higher treatment intensity. By expanding access to formal long-term care services, in-kind benefits may help stabilize health conditions and reduce avoidable hospitalizations, thereby generating a substitution effect from inpatient care toward long-term care services. The absence of a similar effect under the mixed-benefit mode suggests that cash-based support alone may be insufficient to alter hospitalization patterns among disabled elderly individuals.

Effects on healthcare expenditures. The estimated effects on healthcare expenditures are presented in Table 4. Regarding total medical costs, the in-kind benefit model was associated with a substantial and statistically significant reduction, with a DID coefficient of −2597.6 (*p* < 0.001). In contrast, the coefficient for the mixed-benefit mode was negative but not statistically significant. The analysis of out-of-pocket (OOP) expenditures further elucidates the financial impact of the two benefit modes. For total annual OOP payments, the in-kind benefit mode produced a significant reduction, with a coefficient of −667.4 (*p* < 0.001), while the mixed-benefit mode showed a non-significant negative coefficient. When disaggregated by service type, the in-kind mode significantly lowered both outpatient OOP (−198.3, *p* < 0.001) and inpatient OOP (−350.2, *p* = 0.020). By comparison, the corresponding coefficients for the mixed-benefit mode were statistically insignificant and less precisely estimated.

In summary, the baseline results indicate a clear divergence in the effectiveness of the two LTCI benefit designs. The in-kind benefit mode demonstrated robust and statistically significant effects in reducing overall medical costs by RMB 2597.6 per year, lowering out-of-pocket expenditures by RMB 198.3 per year for outpatient care and by RMB 350.2 per year for inpatient care, and decreasing inpatient service utilization. In contrast, the mixed-benefit mode showed limited and inconsistent effects, with a modest reduction in outpatient visits but a statistically insignificant impact on medical costs or inpatient utilization. These findings suggest that the structure of benefit provision, specifically the in-kind delivery of services, plays a critical role in shaping the policy’s impact on healthcare use and financial burden. These contrasting patterns can be understood in light of differences in targeting efficiency and behavioral responses across benefit designs. Under the in-kind benefit mode, LTCI resources are directly tied to the provision of formal care services, which may substitute for hospital-based treatment by addressing daily care needs and preventing health deterioration. This service-based design limits the scope for diversion of benefits to non-medical uses and ensures that support is aligned with beneficiaries’ actual care needs. In contrast, the mixed-benefit mode allows for cash transfers, which may weaken the link between LTCI benefits and healthcare utilization, particularly among elderly individuals facing competing household expenditures or limited capacity to translate cash subsidies into effective care. As a result, the mixed-benefit mode exhibits weaker and less consistent effects on both healthcare utilization and medical expenditures.

As shown in Table 2, the treatment groups (in-kind and mixed) differ from the control group in key baseline characteristics. However, our main DID specification includes province–year fixed effects, individual-level covariates (age, education, health insurance, chronic illness and so on), and pre-trend controls, which jointly absorb time-invariant heterogeneity and evolving confounders. Moreover, the parallel trends test confirms no pre-existing differential trends in outcomes between treatment and control groups. Thus, the identification strategy effectively mitigates their influence, lending credibility to the estimated causal effects reported in Table 4. This aligns with our interpretation that the in-kind benefit’s stronger impact stems from its service-based design rather than pre-existing differences. The following sections further examine the robustness of these estimates and explore potential heterogeneity in these effects across subpopulations.

## 5. Robustness Test Results

### 5.1. Parallel Trend Test

**Figure 1:**
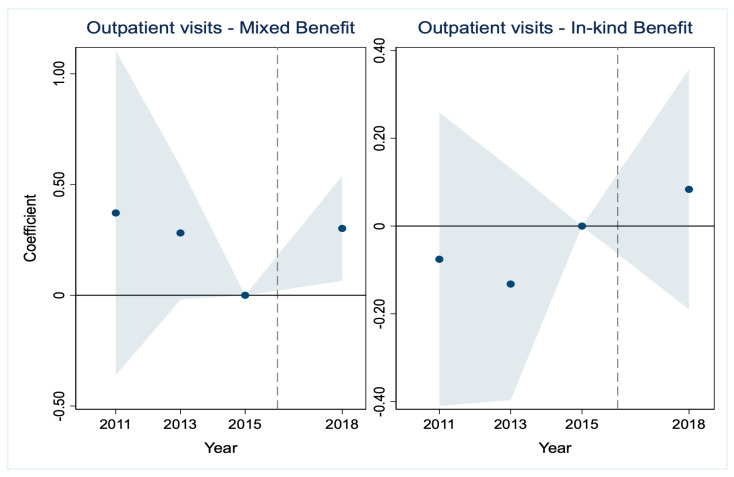
Parallel trends test for outpatient visits. Note: The solid line represents the 95% confidence intervals from the event study analysis. The estimated coefficients for 2011 and 2013 are −0.076 and −0.132, respectively, and both are statistically insignificant.

**Figure 2:**
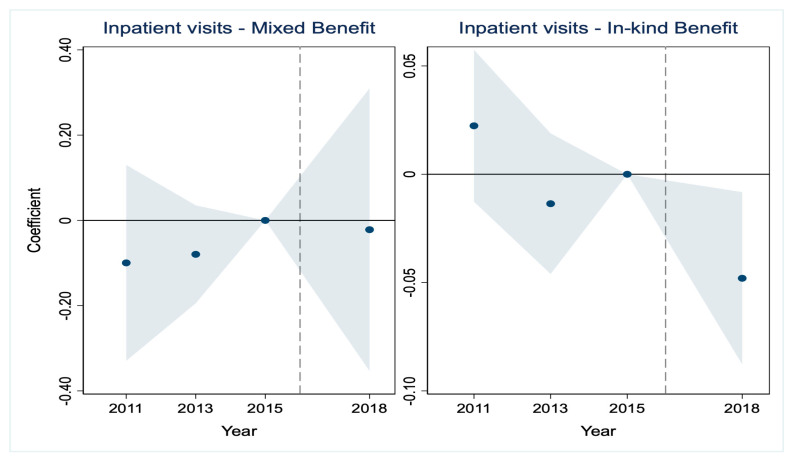
Parallel trends test for inpatient visits. Note: The solid line represents the 95% confidence intervals from the event study analysis. The estimated coefficients for 2011 and 2013 are 0.022 and −0.014, respectively, and both are statistically insignificant.

**Figure 3:**
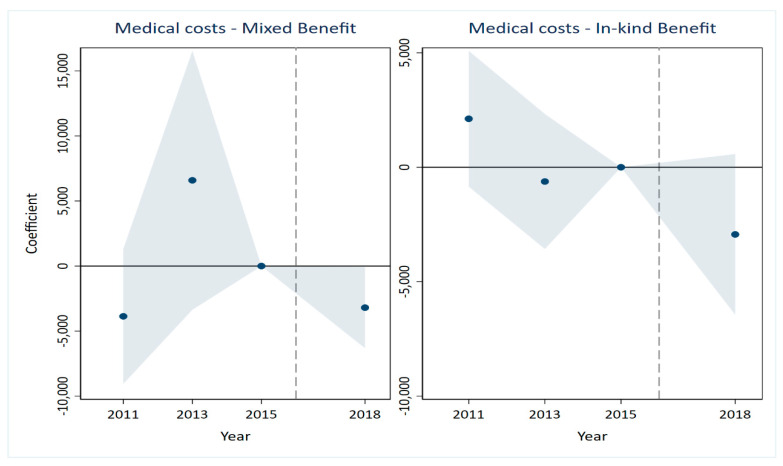
Parallel trends test for total medical costs. Note: The solid line represents the 95% confidence intervals from the event study analysis. The estimated coefficients for 2011 and 2013 are 2118.157 and −623.238, respectively, and both are statistically insignificant.

**Figure 4:**
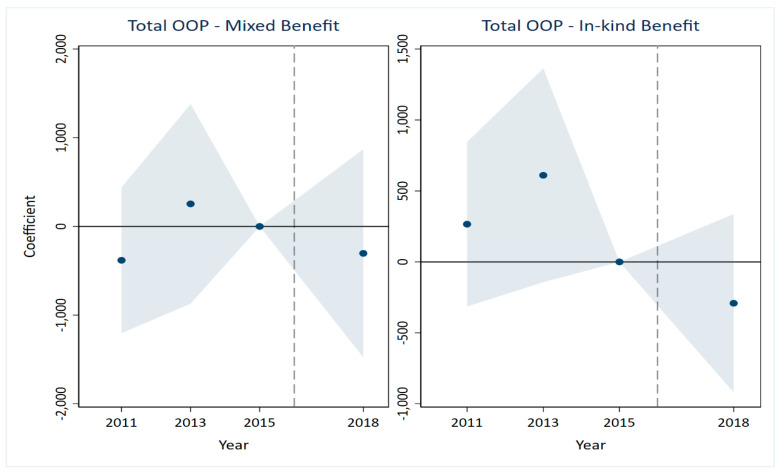
Parallel trends test for total out-of-pocket. Note: The solid line represents the 95% confidence intervals from the event study analysis. The estimated coefficients for 2011 and 2013 are 265.894 and 610.251, respectively, and both are statistically insignificant.

**Figure 5:**
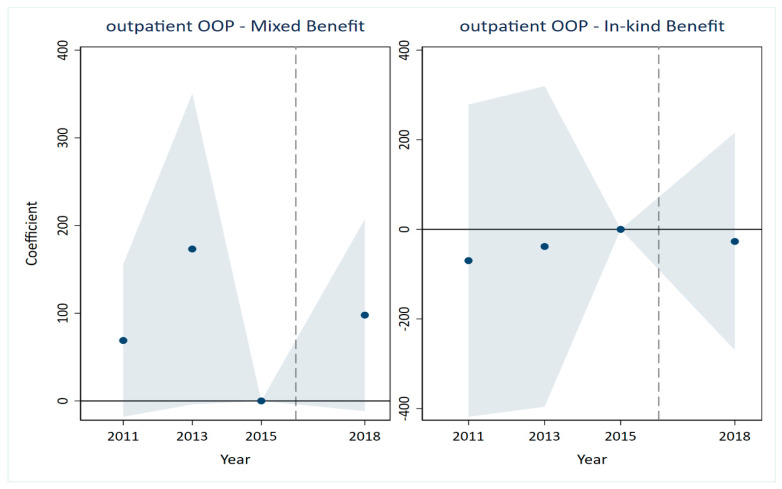
Parallel trends test for outpatient out-of-pocket. Note: The solid line represents the 95% confidence intervals from the event study analysis. The estimated coefficients for 2011 and 2013 are −69.808 and −38.202, respectively, and both are statistically insignificant.

**Figure 6:**
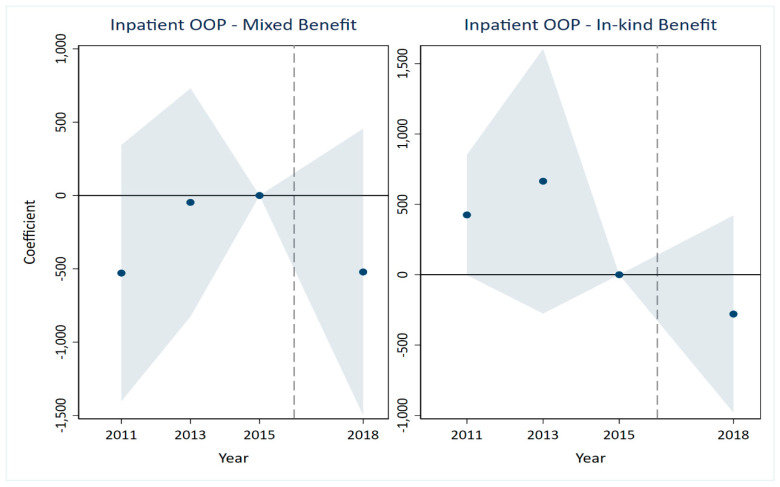
Parallel trends test for inpatient out-of-pocket. Note: The solid line represents the 95% confidence intervals from the event study analysis. The estimated coefficients for 2011 and 2013 are 425.006 and 664.583, respectively, and both are statistically insignificant.

A critical prerequisite for the validity of the DID estimates is that the treatment and control groups would have followed parallel trends in the absence of the LTCI intervention [35,36]. The results are presented in Figure 1, Figure 2, Figure 3, Figure 4, Figure 5 and Figure 6, corresponding to our six main outcome variables: outpatient and inpatient visits, total medical costs, total OOP expenditures, outpatient OOP, and inpatient OOP. Across all figures, the estimated coefficients for the pre-policy years are statistically indistinguishable from zero, with confidence intervals that consistently include zero. This pattern holds for both the mixed-benefit and in-kind benefit groups. The absence of statistically significant differential trends prior to 2016 supports the parallel trends assumption and lends credibility to the causal interpretation of our DID estimates.

Healthcare utilization (Figure 1 and Figure 2). For both outpatient and inpatient visits, the pre-trend coefficients for both benefit groups are statistically insignificant, with confidence intervals that include zero in all pre-2016 years. This consistent pattern across outcomes and groups provides strong support for the parallel trend assumption regarding service utilization.

Healthcare expenditures (Figure 3, Figure 4, Figure 5 and Figure 6). A similar pattern is observed for all expenditure outcomes. The estimated coefficients for total medical costs (Figure 3), total OOP (Figure 4), outpatient OOP (Figure 5), and inpatient OOP (Figure 6) show no statistically significant pre-trends for either the mixed-benefit or in-kind benefit groups in the years preceding the policy rollout. The confidence intervals for these pre-period estimates consistently encompass zero.

We have incorporated the key pre-trend coefficients and their significance levels directly into the event study Figure 1, Figure 2, Figure 3, Figure 4, Figure 5 and Figure 6. In the event study, we use 2015 as the reference year, the last pre-policy wave before most LTCI pilots were launched. This visual presentation offers an intuitive illustration of the parallel trends’ assumption, as none of the pre-treatment coefficients are marked with asterisks, indicating they are all statistically insignificant. The absence of statistically significant differential trends between the treatment and control groups prior to the LTCI implementation validates the key identifying assumption of our DID model. This supports the causal interpretation of our baseline estimates regarding the differential impacts of the two benefit payment modes.

### 5.2. Placebo Test

**Figure 7:**
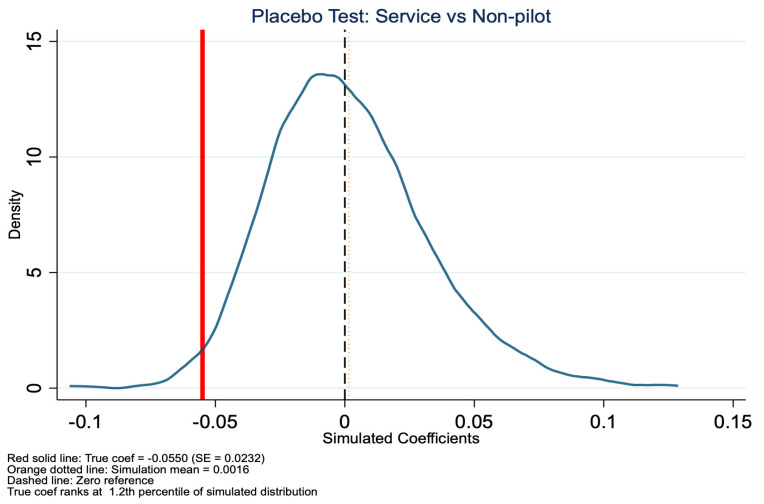
Placebo test (in-kind benefit mode): coefficient distribution for inpatient visits.

**Figure 8:**
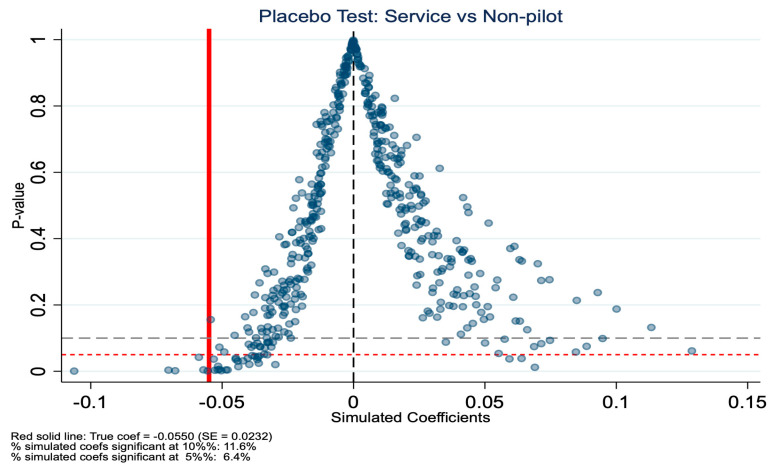
Placebo test (in-kind benefit mode): *p*-values for inpatient visits.

**Figure 9:**
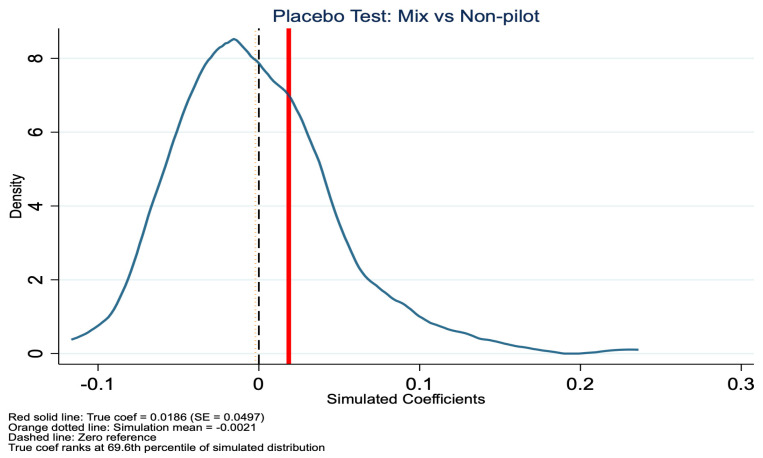
Placebo test (mixed benefit mode): coefficient distribution for inpatient visits.

**Figure 10:**
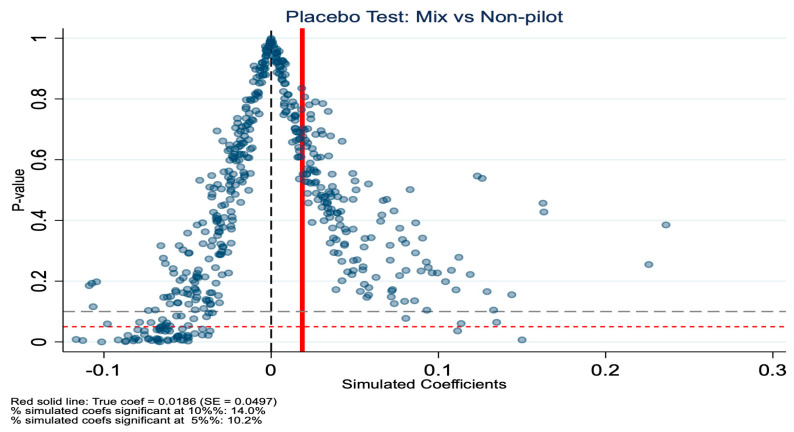
Placebo test (mixed benefit mode): *p*-values for inpatient visits.

To validate the robustness of our baseline DID estimates, we conducted a placebo test. This method involves randomly assigning treatment status and re-estimating the model to examine whether the observed policy effects could arise by chance. For the placebo test, we randomly reassigned treatment status at the city level and repeated the DID estimation 500 times. The results are shown in Figure 7, Figure 8, Figure 9 and Figure 10. Specifically, for the in-kind benefit mode, the true estimated coefficient on inpatient visits is −0.0550 (SE = 0.0232). In the placebo distribution, only 6.4% of simulated coefficients are significant at the 5% level, and the true estimate falls at the 1.2nd percentile, far in the left tail, indicating it is highly unlikely to be due to random factors. In contrast, for the mixed-benefit mode, the true coefficient is 0.0186 (SE = 0.0497), with 10.2% of placebo estimates significant at the 5% level, and the true estimate ranks at the 69.6th percentile of the simulated distribution, well within the null range, consistent with its statistical insignificance in the main analysis. In summary, the placebo test supports our core findings: the in-kind benefit mode has a robust causal effect in reducing inpatient visits, while the mixed-benefit mode shows no significant impact.

### 5.3. Further Robustness Check

We explored alternative model specifications to account for the highly skewed distribution of cost data, which features a large share of zero expenditure, ranging from approximately 50% to 70% across survey waves. We adopted a Two-Part Model (TPM) to handle the semi-continuous nature of medical spending, where a substantial mass of observations is at zero, and the positive values exhibit pronounced skewness. Further details are provided in Appendix A (Table A1 and Table A2). The results from this approach remain consistent with those obtained from our primary specification.

## 6. Heterogeneity Analysis

To further explore whether the effects of different LTCI benefit payment modes vary across population subgroups, we conducted a heterogeneity analysis by household registration status (hukou), distinguishing between rural and urban residents. Hukou status is a particularly relevant dimension in the Chinese context, as it is closely associated with disparities in healthcare access, socioeconomic resources, and baseline health conditions.

Healthcare utilization. Table 5 reports the heterogeneous effects of LTCI benefit payment modes on outpatient and inpatient utilization by hukou status. For outpatient visits, the mixed-benefit mode exhibits a statistically significant reduction only among urban residents (coefficient = −0.149, *p* < 0.1), while the corresponding effect for rural residents is negative but statistically insignificant. In contrast, the in-kind benefit mode significantly reduces outpatient visits among rural residents (coefficient = −0.0707, *p* < 0.01), whereas the effect for urban residents is smaller in magnitude and not statistically significant. A similar pattern is observed for inpatient utilization. The in-kind benefit mode leads to a significant decline in inpatient admissions among rural residents (coefficient = −0.0651, *p* < 0.01), but the estimated effect for urban residents is statistically insignificant. For the mixed-benefit mode, both rural and urban subsamples exhibit a statistically insignificant change in inpatient visits.

Taken together, these findings suggest that in-kind benefits are more effective in reducing healthcare utilization among rural residents, particularly for inpatient services, while the utilization effects of the mixed-benefit mode are limited and largely concentrated among urban residents for outpatient care only.

Healthcare expenditures. Substantial heterogeneity by hukou status is also evident in the effects on healthcare expenditures. As shown in Table 5, the mixed-benefit mode is associated with a significant reduction in total medical costs among rural residents (coefficient = −2169.4, *p* < 0.01), whereas the corresponding estimate for urban residents is positive and statistically insignificant. Similarly, the in-kind benefit mode produces a pronounced reduction in total medical costs among rural residents (coefficient = −3717.6, *p* < 0.01), while the effect among urban residents is smaller and not statistically significant. Regarding OOP expenditures, in Table 5, the in-kind benefit mode demonstrates consistent and statistically significant reductions for both rural and urban residents, although the magnitude is notably larger for urban residents. Specifically, total OOP expenditures decline by 311.4 RMB (*p* < 0.05) among rural residents and by 1464.1 RMB (*p* < 0.01) among urban residents under the in-kind mode. As shown in Table 5, the disaggregated results indicate that outpatient OOP expenditures decrease significantly for both rural (−166.6, *p* < 0.05) and urban residents (−305.3, *p* < 0.01), whereas reductions in inpatient OOP are statistically significant only among urban residents (−842.6, *p* < 0.1). By contrast, the mixed-benefit mode does not generate statistically significant reductions in total or outpatient OOP expenditures for either hukou group, although a modest decline in inpatient OOP is observed among urban residents (−921.8, *p* < 0.1).

The observed heterogeneity by hukou status reflects fundamental differences in baseline healthcare access, economic resources, and patterns of care utilization between rural and urban residents. For rural residents, where access to formal healthcare services is relatively constrained, and unmet care needs are more prevalent, the in-kind benefit mode significantly reduces both outpatient and inpatient utilization as well as total medical costs. This suggests that service-based LTCI benefits effectively alleviate access barriers and reduce reliance on hospital-based care among rural elderly populations. In contrast, urban residents typically exhibit higher baseline levels of healthcare utilization and medical spending.

Accordingly, the primary effect of in-kind benefits in urban areas manifests as a substantial reduction in out-of-pocket expenditures rather than changes in utilization intensity, indicating a stronger financial protection effect. The mixed-benefit mode, however, shows limited effectiveness across both hukou groups, particularly in rural areas, potentially due to insufficient cash benefit levels and weaker links between cash transfers and actual service utilization.

Overall, the heterogeneity analysis reveals clear differences in policy effectiveness across hukou groups. The in-kind benefit mode delivers more robust and consistent effects for rural residents in reducing healthcare utilization and total medical costs, likely reflecting stronger access constraints and better responsiveness to service-based coverage in rural areas. Meanwhile, urban residents experience larger reductions in out-of-pocket expenditures under the in-kind mode, consistent with their higher baseline utilization and spending levels. In contrast, the mixed-benefit mode exhibits limited and uneven effects across hukou groups, with statistically significant impacts confined to a small number of outcomes. The reform’s impacts differ systematically by hukou status under the in-kind benefit model: rural residents experience larger reductions in total healthcare utilization and overall medical expenditures, whereas urban residents see larger declines in out-of-pocket (OOP) payments. Rural residents historically faced more limited access to formal healthcare and weaker financial protection due to their enrollment in less generous rural cooperative schemes (e.g., NCMS), making them more responsive to the expanded in-kind coverage, which directly reduced both utilization barriers and total costs. In contrast, urban residents, who were typically covered by more comprehensive urban employee or resident insurance schemes, already had relatively better access; thus, the primary marginal benefit for them was a reduction in OOP burdens rather than changes in utilization volume.

## 7. Discussion and Conclusions

### 7.1. Limitations and Directions for Future Research

The paper is subject to the following limitations, which may serve as promising directions for future research. First, our analysis employs a standard ADL-based definition of disability that is broader than the formal eligibility criteria, which often require severe disability, used in many LTCI pilot cities. While this enhances the external validity for the general elderly population with care needs, it implies that our estimated effects represent an average across individuals who may or may not qualify for benefits under the official program rules. Second, the CHARLS dataset does not include direct information on individual LTCI enrollment status or actual benefit receipt. As a result, our empirical strategy adopts an Intent-to-Treat (ITT) design, comparing outcomes for all elderly residents in pilot cities to those in non-pilot areas. Consequently, our estimates reflect the net effect of policy availability rather than the impact on confirmed beneficiaries. Third, our primary analysis relies on a four-wave panel (2011, 2013, 2015, 2018), yielding only one post-reform observation. This restricts our capacity to evaluate the long-term dynamic effects of LTCI policies and to fully rule out short-run transitional phenomena. Fourth, it is possible that other local health or social welfare policies were introduced concurrently with the LTCI pilots in our treatment cities. Although our model includes city and year fixed effects to account for time-invariant city characteristics and common temporal trends, unobserved, city-specific policy changes could potentially bias our estimates.

### 7.2. Conclusions

This study provides empirical evidence on the differential effects of two LTCI benefit payment modes—in-kind benefits and mixed benefits—on healthcare utilization and expenditures among middle-aged and older adults in China. Our findings reveal that the design of benefit delivery matters for policy effectiveness.

First, the in-kind benefit mode demonstrated robust and statistically significant effects in reducing healthcare utilization (particularly inpatient visits), total medical costs, and out-of-pocket expenditures. This is consistent with the theoretical expectations that in-kind provision helps ensure resources are directed toward intended healthcare services, minimizing the risk of diversion to non-medical uses. By contrast, the mixed-benefit mode showed limited and inconsistent effects, with only a modest reduction in outpatient visits and no significant impact on inpatient utilization or medical expenditures. These results suggest that the cash subsidy component in the mixed mode may not effectively translate into reduced healthcare costs or altered care-seeking behavior, potentially due to liquidity constraints, information asymmetries, or behavioral factors among elderly beneficiaries.

Second, our heterogeneity analysis reveals important disparities across rural and urban residents. The in-kind benefit mode was particularly effective in reducing healthcare utilization among rural residents, a finding that likely reflects their higher constraints in accessing formal care and higher sensitivity to service-based support. Meanwhile, urban residents experienced larger reductions in out-of-pocket expenditures under the in-kind mode, possibly because of their higher baseline healthcare utilization and expenditure levels. These findings contribute to the growing literature on health equity and social insurance design by highlighting how benefit structures can differentially affect populations with varying socioeconomic resources and healthcare access.

First, our finding that the in-kind model significantly reduces healthcare use and costs aligns with this body of evidence [17,18,19,20,21,22,23], reinforcing the view that direct service delivery enhances care coordination and substitutes for acute medical interventions. Our study aligns closely with Lei and Zhang’s [24] findings in identifying a “cash-out puzzle” in China’s LTCI system: both papers demonstrate that in-kind benefits improve elderly welfare, whereas cash or mixed (cash-dominated) benefits yield statistically insignificant or markedly weaker effects. This convergence underscores the critical role of benefit design in contexts where beneficiaries may lack the capacity or support to effectively convert unrestricted cash into quality long-term care.

A small but growing body of literature examines the differential effects of long-term care (LTC) benefit designs, primarily in high-income settings. Notably, two studies from Spain, Costa-Font et al. [21] and Costa-Font et al. [37], find that cash allowances for informal caregiving reduce hospital use and increase both informal care receipt and intergenerational cash transfers. While both in-kind services and cash benefits were associated with reduced hospital admissions, the decline was smaller under cash, a pattern that aligns with our finding that in-kind benefits generate stronger healthcare utilization effects than mixed (cash-dominated) models. However, an important distinction emerges in the context and mechanism. The Spanish studies assume a well-functioning LTC market and relatively high beneficiary capacity to deploy cash effectively. The conditions differ markedly from those in rural China, where limited infrastructure and lower health literacy may constrain the ability of cash recipients to translate transfers into quality care. In our setting, the mixed-benefit model shows no statistically significant reduction in healthcare use, suggesting that cash alone may be insufficient without complementary support systems.

Second, there are several mechanisms for weaker mixed-benefit effects, namely, (i) diversion of cash to non-medical uses, (ii) limited health and long-term care literacy, and (iii) persistent access barriers in rural areas to our observed rural–urban heterogeneity. Specifically, we argue that rural residents face steeper constraints in translating cash into effective care due to underdeveloped local LTC markets and lower capacity to navigate complex care decisions. Urban residents, while better positioned, still exhibit limited responses under the mixed model since unrestricted cash may be used for consumption smoothing rather than targeted care investment. This explains why only the in-kind model, which directly supplies vetted services, generates significant reductions in downstream healthcare utilization, especially among rural hukou holders.

Our results carry important implications for LTCI policy design in China and other aging societies. First, policymakers may wish to consider prioritizing in-kind benefit provision over mixed or cash-based models when the goal is to reduce unnecessary healthcare utilization and alleviate financial burdens. The stronger performance of in-kind benefits in our study suggests this mode may be more effective in targeting resources to actual care needs. Second, the heterogeneity findings point to the potential value of benefit designs that account for regional and demographic differences. For instance, in-kind benefits could be especially beneficial in rural areas where access barriers are more pronounced, while urban areas might benefit from complementary measures to address high out-of-pocket costs.

This study suggests that the choice between in-kind and mixed benefit payment modes under China’s LTCI system can influence healthcare utilization and expenditures among elderly and disabled populations. The in-kind benefit mode emerges as potentially more effective in reducing both service use and financial burden, particularly for vulnerable subgroups such as rural residents. These findings underscore the importance of aligning benefit design with policy objectives and population needs. As countries worldwide grapple with the challenges of population aging, evidence-based insights into social insurance design, such as those provided here, will be crucial for developing sustainable and equitable long-term care systems.

## Figures and Tables

**Table 1:** LTCI pilot cities and their benefit payment modes.

Province	City	Benefit Payment Modes
Jiangxi	Shangrao	Mixed
Sichuan	Chengdu	Mixed
Hubei	Jingmen	Mixed
Jiangsu	Xuzhou	Mixed
Shandong	Jinan	In-kind
Jilin	Jilin	In-kind
Hebei	Chengde	In-kind
Shanghai	Shanghai	In-kind
Anhui	Anqing	In-kind
Guangdong	Guangzhou	In-kind
Shandong	Linyi	In-kind
Heilongjiang	Qiqihar	In-kind
Jiangsu	Suzhou	In-kind
Zhejiang	Ningbo	In-kind

Note: The long-term care insurance system offers two benefit types: mixed, in which beneficiaries can choose either in-kind services or cash payments, and in-kind, which provides in-kind benefits only.

**Table 2:** Descriptive statistics of key variables by LTCI benefit payment mode.

Variables	Treatment Group (In-Kind)		Treatment Group (Mixed)		Control Group	
	Mean	SD	Mean	SD	Mean	SD
Outpatient Visits	0.19	0.71	0.23	0.66	0.41	1.40
Inpatient Visits	0.11	0.69	0.17	0.63	0.22	0.91
Medical Costs	2396.3	7685.1	2679.1	9716.8	4106.7	16,914.9
Total OOP	663.5	4281.6	378.3	1717.8	1135.6	7756.9
Outpatient OOP	45.4	257.8	105.6	555.6	174.7	1796.2
Inpatient OOP	620.5	4261.7	275.2	1390.5	980.3	7338.8
Age	55.7	9.50	56.1	8.77	60.1	10.5
Education Level	2.69	1.12	2.60	1.15	2.01	1.05
Marital Status	0.94	0.24	0.94	0.24	0.86	0.34
Health Insurance	1	0	1	0	0.95	0.22
Pension Insurance	0.51	0.50	0.55	0.50	0.50	0.50
Hukou (1 = rural)	0.53	0.50	0.49	0.50	0.77	0.42
Number of Children	1.54	0.85	1.97	1.11	2.61	1.41
Consumption Level	19,119.6	33,073.1	17,131.6	18,137.0	13,987.9	25,868.6
Chronic Illness	0.46	0.50	0.58	0.50	0.75	0.43
Inpatient Treatment	0.063	0.24	0.094	0.29	0.14	0.35
Observations	396		127		210,167	

Notes: OOP represents the out-of-pocket expenditure. All outcomes of medical costs are measured in RMB per year. The treatment group (In-kind) includes individuals in LTCI pilot cities who receive only in-kind services, while the treatment group (Mixed) includes individuals in LTCI pilot cities who receive a combination of in-kind services and cash benefits. The control group consists of individuals from non-pilot cities who are not covered by LTCI.

**Table 3:** Effect of LTCI benefit modes on outpatient visits and inpatient visits.

	(1)	(2)	(3)	(4)
Variables	Outpatient Visits		Inpatient Visits	
	Mixed	In-Kind	Mixed	In-Kind
Mixed # post	−0.226 *		0.0186	
	(0.119)		(0.0497)	
In-kind # post		−0.0714 *		−0.0550 **
		(0.0411)		(0.0232)
Observations	33,161	35,375	33,490	35,716
R-squared	0.458	0.460	0.760	0.752

Note: All regression specifications control for age, education level, marital status, health insurance coverage, pension insurance status, hukou type, consumption level, and number of children, and other covariates, and include both individual and year fixed effects. Cluster-robust standard errors are shown in parentheses and are clustered at city level. Asterisks reflect significance level: ** *p* < 0.05, * *p* < 0.1. The notation ‘#’ denotes the interaction term, constructed as the product of the two variables.

**Table 4:** Effect of LTCI benefit modes on medical expenditures.

	(1)	(2)	(3)	(4)	(5)	(6)	(7)	(8)
Variables	Medical Costs		Total OOP		Outpatient OOP		Inpatient OOP	
	Mixed	In-Kind	Mixed	In-Kind	Mixed	In-Kind	Mixed	In-Kind
Mixed # post	−1071.7		−352.9		62.27		−405.5	
	(895.7)		(500.1)		(49.42)		(535.5)	
In-kind # post		−2597.6 ***		−506.0 ***		−198.3 ***		−350.2 **
		(599.5)		(188.8)		(53.29)		(150.1)
Observations	33,500	35,726	33,500	35,726	32,887	35,048	33,158	35,375
R-squared	0.455	0.455	0.494	0.493	0.439	0.424	0.494	0.493

Note: All outcomes of medical cost are measured in RMB per year. All regression specifications control for age, education level, marital status, health insurance coverage, pension insurance status, hukou type, consumption level, number of children, and other covariates, and include both individual and year fixed effects. Cluster-robust standard errors are shown in parentheses and are clustered at city level. Asterisks reflect significance level: *** *p* < 0.01, ** *p* < 0.05. The notation ‘#’ denotes the interaction term, constructed as the product of the two variables.

**Table 5:** Heterogeneous effects of LTCI benefit payment modes on healthcare utilization and healthcare expenditures by hukou status.

	Mixed Benefit Mode		In-Kind Benefit Mode	
Variables	Rural	Urban	Rural	Urban
Outpatient Visits	−0.274	−0.149 *	−0.0707 ***	−0.104
	(0.199)	(0.0848)	(0.0257)	(0.0904)
Inpatient Visits	0.0599	−0.0704	−0.0651 ***	−0.0279
	(0.0393)	(0.0471)	(0.0241)	(0.0346)
Total Medical Costs	−2169.4 ***	1625.3	−3717.6 ***	−790.4
	(554.1)	(1834.7)	(460.6)	(940.4)
Total OOP	−127.1	−820.2	−311.4 **	−1464.1 ***
	(669.7)	(583.8)	(152.8)	(557.9)
Outpatient OOP	74.46	1.227	−166.6 **	−305.3 ***
	(56.54)	(60.29)	(69.60)	(64.39)
Inpatient OOP	−194.4	−921.8 *	−146.2	−842.6 *
	(736.1)	(546.6)	(113.5)	(471.6)

Note: All regression specifications control for age, education level, marital status, health insurance coverage, pension insurance status, hukou type, consumption level, and number of children, and other covariates, and include both individual and year fixed effects. Cluster-robust standard errors are shown in parentheses and are clustered at city level. Asterisks reflect significance level: *** *p* < 0.01, ** *p* < 0.05, * *p* < 0.1.

## Data Availability

The data presented in this study are available from the CHARLS at https://charls.pku.edu.cn/.

## Abbreviations

LTCI	long-term care insurance
CHARLS	China Health and Retirement Longitudinal Study
DID	difference-in-differences
ADL	activities of daily living
OOP	out-of-pocket

## Appendix A

We examined alternative specifications to address the highly skewed nature of cost data, which includes a substantial proportion of zero expenditures (approximately 50–70% across waves). We employed a Two-Part Model (TPM) to address the semi-continuous nature of medical expenditures, characterized by a mass of zero observations and a skewed distribution of positive costs. The first part estimates the probability of incurring any expenditure (the probability of outpatient/inpatient cost), while the second part estimates the conditional intensity of expenditures using log(cost) for positive expenses. The results remain consistent with our primary findings: in-kind LTCI benefit design significantly reduces out-of-pocket spending, whereas mixed benefits show no significant effect. This approach aligns with recommendations in the literature for handling zero-inflated right-skewed health expenditure data.

**Table A1:** Effect of LTCI benefit modes on the probability of outpatient and inpatient.

	(1)	(2)	(3)	(4)
Variables	Probability of Outpatient		Probability of Inpatient	
	Mixed	In-Kind	Mixed	In-Kind
Mixed # post	0.00432		0.000738	
	(0.0182)		(0.00112)	
In-kind # post		−0.0276 *		−0.000351 ***
		(0.0148)		(0.000109)
Observations	47,404	50,426	33,490	35,716
R-squared	0.430	0.430	0.760	0.752

Note: All regression specifications control for age, education level, marital status, health insurance coverage, pension insurance status, hukou type, consumption level, and number of children, and other covariates and include both individual and year fixed effects. Cluster-robust standard errors are shown in parentheses and are clustered at city level. Asterisks reflect significance level: *** *p* < 0.01, * *p* < 0.1. The notation ‘#’ denotes the interaction term, constructed as the product of the two variables.

**Table A2:** Effect of LTCI benefit modes on medical expenditures (logarithm cost).

	(1)	(2)	(3)	(4)	(5)	(6)
Variables	Total OOP		Outpatient OOP		Inpatient OOP	
	Mixed	In-Kind	Mixed	In-Kind	Mixed	In-Kind
Mixed # post	0.00470		−0.0649		0.122	
	(0.232)		(0.226)		(0.661)	
In-kind # post		−0.333 **		−0.612 ***		−0.812 **
		(0.127)		(0.121)		(0.409)
Observations	7332	7626	4369	4569	2275	2312
R-squared	0.772	0.772	0.614	0.612	0.604	0.608

Note: All regression specifications control for age, education level, marital status, health insurance coverage, pension insurance status, hukou type, consumption level, and number of children, and other covariates and include both individual and year fixed effects. Cluster-robust standard errors are shown in parentheses and are clustered at city level. Asterisks reflect significance level: *** *p* < 0.01, ** *p* < 0.05. The notation ‘#’ denotes the interaction term, constructed as the product of the two variables.
